# Castration of dromedary camel through prescrotal midline incision

**Published:** 2012-10-19

**Authors:** M.N. Telfah, M.I. Siddiqui, S.A. Taleb

**Affiliations:** *Central Veterinary Hospital, Al-Wathba, Abu Dhabi, United Arab Emirates*

**Keywords:** Camel, Castration, Prescrotal, Transfixation

## Abstract

A total of 165 camels of different ages were castrated through a small, prescrotal midline incision between January, 2010 and December, 2011. The incision was closed with one interrupted, horizontal mattress suture using USP-2 chromic catgut. In 14/165 animals (8.5%) postoperative infection (sepsis) developed, which healed in two to three weeks after open wound management. The remaining 151 animals had an uneventful recovery, but a slight edematous swelling of the scrotum was observed in 8 of the 151 animals (5.3%), which was self-limiting and of no significance. No primary or secondary postoperative bleeding was noticed in any of the animals. It was concluded that this technique was less time consuming with negligible postoperative care and complications when performed under standard surgical principles.

## Introduction

Testes in the camel are ovoid in shape and are located in the perineal position. At the age of about 7 months, they lie caudally to the superficial inguinal ring and usually descend in the scrotum by second to third year of animal’s life.

At the time of descent, they are quite small but increase in size at the onset of puberty (Smuts and Bezuidenhout, 1987). It has been noticed that they become enlarged and protrude when the animal is sexually active in the rutting season and return to their normal size in the sexually dormant period. Usually one testicle is higher in position, and in the vast majority of animals the right testicle is smaller than the left.

Castration of camels has been a common practice since decades (Droandi, 1920, 1939). Castration is an elective procedure which is normally done to render the camel docile and easily controllable. Sometimes, castration is done to avoid accidental mating when the animal is not desirable for breeding purpose and in certain testicular abnormalities such as orchitis, irreparable traumatic injuries, tumors etc. Furthermore, castration is done as an elective procedure in almost all the animal species for the same purpose.

The standard surgical procedure involves open castration in which both scrotal sacs and the tunica vaginalis cavities are opened through two large incisions, the testes removed and the wounds left open for postoperative care until complete healing takes place (Silvey, 1978; Thomson, 1978).

Castration of camels in the standing position has been reported by Tibary and Anouassi (2004). Primary bleeding may be a potential complication if the vascular portion of the spermatic cord is not properly ligated or emasculated and in cases where the surgical procedure is not performed according to the standard surgical principles, infection of the surgery site may take place necessitating meticulous postoperative care (Nelson, 1987). In rare instances, formation of unilateral or bilateral scirrhous cord as a result of chronic infection may necessitate an elaborate second surgical procedure (Siddiqui and Telfah, 2010).

In our experience; unlike that in the horse, there is negligible postoperative drainage in the camel after castration. Hence, we have been doing this procedure through two small scrotal incisions which were just enough to squeeze out the testicle followed by closure of each incision with one interrupted, horizontal mattress suture using USP-2 chromic catgut. This precluded the needed postoperative care of the case compared to when the scrotal wounds were left open. However, lately we modified our technique and instead of two incisions; a single, small prescrotal midline incision was used to exteriorize both the testicles through it and the incision was closed with one interrupted horizontal mattress suture of USP-2 chromic catgut.

This procedure gave us quite encouraging results; was also cosmetically pleasing, as there was only a small scar in the prescrotal midline region which was even not visible due to its location.

At present we are practicing only this technique of castration in the camel. The report covers the results of 165 cases of castration performed with this technique during a period of 2 years from January, 2010 to December, 2011 and is being presented for consideration and comments by our colleagues engaged in camel practice.

## Surgical Technique

### Control and Anesthesia

Each animal was kept fasting for 24 hours prior to surgery and just before operation, was controlled in the sternal recumbency and was deeply sedated with a mixture of 2% solution of xylazine hydrochloride and 10% solution of ketamine hydrochloride given intravenously at the dose rate of 0.4 mg / Kg of body weight of each drug (Peshin *et al.*, 1980; Trim, 1981). Both the drugs were mixed in the same syringe.

The animal was then put in the left lateral recumbency with the upper hind limb pulled forward and tied securely with the upper fore limb that gave a reasonable space for surgical manipulations. The operation site was thoroughly scrubbed with povidine iodine according to the standard surgical principles and was cleanly dried.

### Operative Steps

1. The upper (right) testicle was pushed forward towards the midline in the prescrotal region and was immobilized there by firmly grasping it between the fingers and thumb of the left hand. A small incision was then made on the midline with the right hand which was just enough to squeeze out the testicle. In case of a small sized testicle, as seen in the young animals of 2-3 years of age; it was difficult to perfectly stabilize it with a single hand. In such a situation, the assistant immobilized the organ with his both hands while the operator made a small incision exactly on the midline to exteriorize the testicle (Fig. 1).

**Fig.1:**
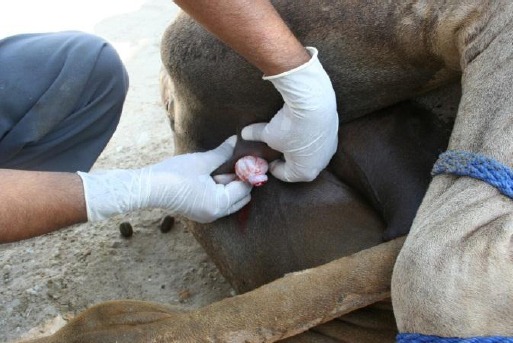
Squeezing of the testicle through a small, prescrotal midline incision.
2. After exteriorizing the testicle, the tunica vaginalis was freed from its attachments as high up as possible and incised longitudinally with an operating scissors.
3. The vascular and non-vascular portions of the spermatic cord were separated from each other through the mesorchium.
4. The non-vascular portion was securely transfixed with USP-2 chromic catgut and severed distal to the transfixation.
5. The vascular portion was maximally exteriorized and was first simply ligated with USP-2 chromic catgut and then transfixed distal to the ligature using the same suture material and was severed below the transfixation ligature (Fig. 2). This technique provided a full security against slippage of the ligature.

**Fig.2:**
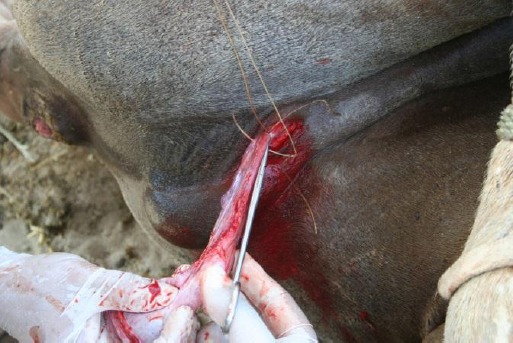
Simple ligation and transfixation of the vascular portion of the spermatic cord with USP-2 chromic catgut.
6. The lower (left) testicle was then pushed towards the incision in the prescrotal region, slightly incised and squeezed out. The spermatic cord was ligated and severed the same way.
7. The skin incision was cleaned with a sterile gauze swab and closed with one interrupted, horizontal mattress suture using USP-2 chromic catgut (Fig. 3). The reason for using catgut to suture the skin incision was that it did not need to be removed.

**Fig.3:**
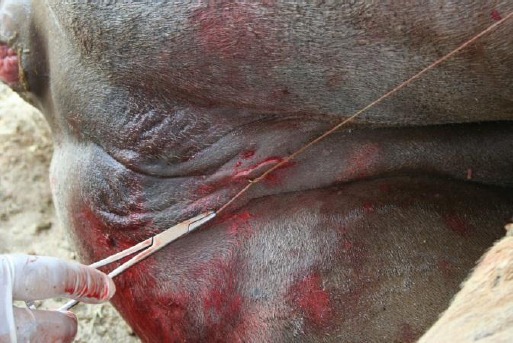
Closure of the skin incision with interrupted horizontal mattress suture using USP-2 chromic catgut.

## Results and Discussion

Out of 165 animals castrated with the prescrotal midline incision technique, only 14 animals experienced the complication of postoperative infection but there was no complaint of primary or secondary postoperative bleeding in any of the animals.

The postoperative infection can be expected, as perfect aseptic technique as described by Nelson (1987) is difficult, rather impossible to follow in the field conditions. The results also indicated that the incidence of postoperative infection increased with the advancement of age (Table 1).

**Table 1:** Postoperative complications in different age groups of animals.

Age group (years)	Number of animals	Postoperative Bleeding	Postoperative Infection	Infection Percentage
3-5	57	Nil	2	3.5 %
5-10	83	Nil	7	8.5 %
>10	25	Nil	5	20 %

Total	165	Nil	14	8.5 %

The increased incidence of postoperative infection with the advancing age may most probably be explained in terms of decreased resistance of the animals to infection with increasing age.

In these animals, the skin sutures were removed and the incisions were slightly enlarged for proper cleaning and dressing of the wounds till complete recovery. The wounds in all of these animals healed in two to three weeks.

The complication of postoperative infection in open method of castration has also been reported by Droandi (1939) and Silvey (1978), with the main reason being that the open scrotal wounds are more prone to contamination and infection.

In contrast, the incidence of postoperative infection was much less in the prescrotal midline incision technique, as there were practically no chances of contamination and infection of the wound in the postoperative period due to its being sutured and prescrotal.

Therefore, the postoperative infection could be attributed to contamination of the surgery site during the surgical procedure as also documented by Thomson (1978).

**Fig.4:**
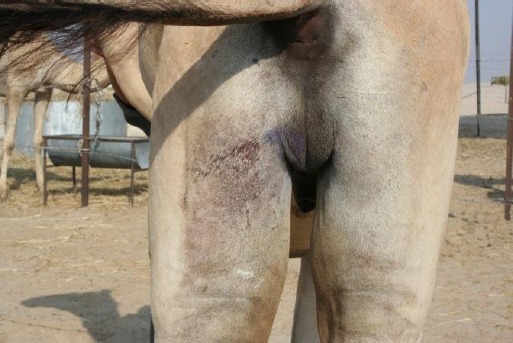
The animal 3 days post-castration. Note cosmetic look of the scrotal region.

None of the animals in this study had the complaint of primary or secondary bleeding, as the vascular portion of the spermatic cord was first ligated and then transfixed; thereby excluding any chances of slippage of the ligatures. The scrotal area presented quite a cosmetic and tidy look (Fig. 4).

It was concluded that if the procedure is performed observing the general surgical principles, the complication of postoperative sepsis can be largely avoided. In addition, the camel being the desert animal; still seems to be quite resistant to low grade infections and this fact goes in favor the surgeon even in the absence of an optimal surgical technique.
